# An initiative of cooperation in Zika virus research: the experience of the ZIKABRA study in Brazil

**DOI:** 10.1186/s12889-021-10596-0

**Published:** 2021-03-23

**Authors:** Silvana Pereira Giozza, Ximena Pamela Díaz Bermúdez, Edna Oliveira Kara, Guilherme Amaral Calvet, Ana Maria Bispo de Filippis, Marcus Vinícius Guimarães Lacerda, Camila Helena Aguiar Bôtto-Menezes, Marcia da Costa Castilho, Rafael Freitas Oliveira Franca, Armando Menezes Neto, Casey Storme, Noemia S. Lima, Kayvon Modjarrad, Maria Cristina Pimenta de Oliveira, Gerson Fernando Mendes Pereira, Nathalie Broutet, on behalf of ZIKABRA Study Team, André Luiz de Abreu, Ximena Pamela Díaz Bermúdez, Camila Helena Aguiar Bôtto-Menezes, Patrícia Brasil, Carlos Alexandre Antunes Brito, Nathalie Jeanne Nicole Broutet, Guilherme Amaral Calvet, Marcia Costa da Castilho, Tatiana Jorge Fernandes, Ana Maria Bispo de Filippis, Rafael Freitas Oliveira Franca, Silvana Pereira Giozza, Ndema Habib, Edna Oliveira Kara, Marcus Vinicius Guimarães Lacerda, Sihem Landoulsi, Morganna Costa Lima, Noemia Lima, Maeve Brito de Mello, Robyn Meurant, Kayvon Modjarrad, Armando Menezes Neto, Gerson Fernando Mendes Pereira, Cristina Pimenta, Casey Storme, Ute Ströher, Anna Thorson, Lydie Trautman

**Affiliations:** 1 Department of Chronic Condition Diseases and Sexually Transmitted Infections, Health Surveillance Secretariat, Ministry of Health, Brazil, SRTVN Quadra 701, Lote D, Edifício PO700 – 5° andar, Brasília, Distrito Federal 70719-040 Brazil; 2 Public Health Department, University of Brasilia, Brasilia, Brazil; 3 World Health Organization, Geneva, Switzerland; 4 Evandro Chagas National Institute of Infectious Diseases, Rio de Janeiro, Brazil; 5 Oswaldo Cruz Institute, Rio de Janeiro, Brazil; 6 Instituto Leônidas & Maria Deane, Fiocruz, Manaus, Brazil; 7 Tropical Medicine Foundation Dr Heitor Vieira Dourado, Manaus, Brazil; 8 Amazonas State University, Manaus, Brazil; 9 Oswaldo Cruz Foundation - Institute Aggeu Magalhães, Recife, Brazil; 10 Henry M. Jackson Foundation for the Advancement of Military Medicine, Bethesda, MD USA; 11 Emerging Infectious Diseases Branch, Walter Reed Army Institute of Research, Silver Spring, MD USA

**Keywords:** Zika virus, Outbreak, Public health emergency, International cooperation in health

## Abstract

**Background:**

The Zika virus outbreak has triggered a set of local and global actions for a rapid, effective, and timely public health response. A World Health Organization (WHO) initiative, supported by the Department of Chronic Condition Diseases and Sexually Transmitted Infections (DCCI) of the Health Surveillance Secretariat (SVS), Brazil Ministry of Health (MoH) and other public health funders, resulted in the start of the “Study on the persistence of Zika virus in body fluids of patients with ZIKV infection in Brazil – ZIKABRA study”. The ZIKABRA study was designed to increase understanding of how long ZIKV persists in bodily fluids and informing best measures to prevent its transmission. Data collection began in July 2017 and the last follow up visit occurred in 06/26/2020.

**Methods:**

A framework for the ZIKABRA Cooperation initiative is provided through a description and analysis of the mechanisms, strategies and the ethos that have guided the models of international governance and technical cooperation in health for scientific exchange in the context of a public health emergency. Among the methodological strategies, we included a review of the legal documents that supported the ZIKABRA Cooperation; weekly documents produced in the meetings and working sessions; technical reports; memorandum of understanding and the research protocol.

**Conclusion:**

We highlight the importance of working in cooperation between different institutional actors to achieve more significant results than that obtained by each group working in isolation. In addition, we point out the advantages of training activities, ongoing supervision, the construction of local installed research capacity, training academic and non-academic human resources, improvement of laboratory equipment, knowledge transfer and the availability of the ZIKABRA study protocol for development of similar studies, favoring the collective construction of knowledge to provide public health emergency responses. Strategy harmonization; human resources and health services; timing and recruiting particularities and processing institutional clearance in the different sites can be mentioned as challenges in this type of initiative.

## Background

The sudden emergence of the Zika virus (ZIKV) in Brazil, the wide geographical range of the mosquito vector of the virus and the link to microcephaly and other important disorders, triggered a call for a global response. On November 11, 2015, the Brazilian Ministry of Health (MoH) declared the ZIKV epidemic as a Public Health Emergency of National Importance [1, 2] and, in February 2016, the World Health Organization (WHO) declared the epidemic to be a Public Health Emergency of International Concern (PHEIC) [3]. Considering the extent of the infection in Brazil and its complications, urgent and coordinated public health strategies were developed in the areas of surveillance, by adopting vector control measures and strong risk communication strategies, in addition to research and development actions in the clinical, laboratory and social areas, summarized in the Brazilian Plan for Fighting *Aedes aegypti* and microcephaly [4]. In turn, the WHO has set out a strategic response plan, the *Strategic Response Framework* (SRF) [5], aiming to foster early responses to the ZIKV emergency, including implementation of clinical research studies, the design of study protocols for diagnosis, the development of studies with qualitative methodologies, the formulation of strategies to reduce the risks of exposure to the *Aedes* mosquito, the development of safe and effective therapies and vaccines and the identification of funding sources to support this extensive research and development agenda.

The “Study on the persistence of Zika virus (ZIKV) in body fluids of patients with ZIKV infection in Brazil – ZIKABRA Study” was conducted to address one of the research gaps identified by the WHO Research Agenda which comprises the characterization of the ZIKV infection [6]. The aim of the study was to assess the presence and duration of Zika virus and related markers in infected individuals and their symptomatic or asymptomatic household contacts [7]. The study was supported by the Department of Chronic Condition Diseases and Sexually Transmitted Infections (DCCI) of the Health Surveillance Secretariat (SVS), Ministry of Health (MoH).

The ZIKABRA study brought together the expertise of a multi-disciplinary group of scientists to meet the requirements of designing a research protocol with complex clinical, epidemiological, biomedical, virological, laboratory and public policy features. The national epidemiological situation and research capacity dictated the selection of the study sites, notably in three major capitals in the northern (Manaus), northeastern (Recife) and southeastern (Rio de Janeiro) regions of the country, with its reference laboratories at the Tropical Medicine Foundation Dr. Heitor Vieira Dourado (FMT-HVD), Oswaldo Cruz Foundation, in Rio de Janeiro (Fiocruz-RJ) and Brazil Oswaldo Cruz Foundation-Institute Aggeu Magalhães, Recife (CPqAM – Fiocruz - PE). On the international side, the United States Department of Defense laboratory, and the Walter Reed Army Institute of Research (WRAIR) provided technical, material and immunologic assay support.

This paper provides a framework for the ZIKABRA initiative through a description and analysis of the mechanisms, strategies and the ethos that have guided the models of international cooperation and scientific exchange between Brazil and international organizations in the context of a public health emergency.

## Methods

The project was jointly funded by the MoH/SVS/DCCI (grant 837059/2016, Process SEI 25000162039201616); WHO (UNDP-UNFPA-UNICEF-WHO-World Bank Special Programme of Research, Development and Research Training in Human Reproduction); WRAIR - (0130602D16) - Cooperation Agreement (W81XWH-18-2-0040) between Henry M. Jackson Foundation for the Advancement of Military Medicine and US Department of the Army; Wellcome Trust (WT) (grant 206522/Z/17/Z); and National Institutes of Health (NIH) (Award Number R21AI139777), thus completing the ZIKABRA international cooperation.

This is a descriptive and documentary analytical study, based on primary data, produced by the ZIKABRA Cooperation. The documents produced by the research study group and technical reports were reviewed. Basically, the weekly meetings fed the information about the management and governance information, once all the representatives of the participant institutions attend these calls.

Data underlying the study cannot be made publicly available due to ethical concerns, as data contain several personally identifiable information. Data are available from Oswaldo Cruz Foundation for researchers who meet the criteria for access to confidential data. To contact information, please report the Declarations section of the Manuscript, Availability data and materials.

### ZIKABRA study: role of partners

The study design was developed in partnership by a team of scientists from WHO, Fiocruz-RJ, CPqAM - Fiocruz-PE, FMT-HVD, WRAIR and DCCI and marked the beginning of the formation of the network of collaborators from MoH, PAHO, WHO, WRAIR, Fiocruz, WT and NIH, which is named ZIKABRA Cooperation.

In 2016, at the time of the ZIKABRA study development, 216,207 probable ZIKV cases were reported in Brazil [8]. From 2015 to 2016, 10,232 suspected cases and 2,202 confirmed cases of microcephaly were reported in the country [9]. The ZIKABRA study [7] is an observational cohort of men and non-pregnant women, aged 18 years or older, with infection confirmed by laboratory tests on blood or urine. Specimens of blood, semen, vaginal secretion, urine, rectal swab, sweat, saliva and breast milk were collected and tested for ZIKV RNA at the partner health facilities through 17 visits distributed over 12 months of follow-up [7]. The criteria used to select the recruitment sites were: high population density; high circulation of ZIKV; strong community health network; laboratory facilities capable of carrying out viral culture, ZIKV antigen assays, RT-PCR, IgM/IgG, neutralizing antibody test (specific for ZIKV, dengue and chikungunya) and virus genetic sequencing [7].

The laboratory component of the ZIKABRA study is a key part of the protocol, whose performance complexity requires mature operational and logistic support, essential to meet the requirements of all steps of the study, which included: recruitment; collection and transport of biological samples and specimens for laboratory testing at partner institutions in Brazil and WRAIR; follow-up of participants; data management and analysis; and production and progressive dissemination of knowledge generated within the scope of the project, [7, 10–12]. The main functions assumed by the institutions that make up the ZIKABRA Cooperation are briefly outlined below.

#### Ministry of Health (MoH)

The research agenda integrated into the Brazilian Plan for Fighting *Aedes aegypti* and Microcephaly [13] guided the building of researcher teams, consortia, agreements, and technical cooperation around a common theme [14], covering all fields of scientific knowledge. The SVS/MoH played a key role in this knowledge-building process by capturing and allocating the necessary financial resources to promote research related to ZIKV and microcephaly. The ZIKABRA study was included in the SVS list of priority studies, in the form of direct contracting [15]. It was left to the discretion of DCCI/SVS/MoH to lead the collaboration process with the WHO and national and international partner institutions. The General Coordination for the Development of Epidemiology in Services (CGDEP/SVS) monitors these studies and promotes the integration between scientific research and health surveillance management, the General Coordination of Public Health Laboratories (CGLAB/SVS) provides laboratory support and, together with DCCI/SVS, participate in the ZIKABRA Cooperation governance.

#### World Health Organization (WHO)

The WHO provided the initial funding, concept and planning for the investigation of ZIKV persistence in body fluids, and was responsible for bringing together funding partners, (WRAIR and WT), and providing the research protocol using the “Ebola RNA Persistence in Semen of Ebola Virus Disease Survivors” [16] as a reference template for the design of the ZIKABRA study. The role of the WHO was particularly crucial for scientific development and in responding to knowledge gaps around new health threats, in defining best practices in prevention and care and in the international dimension of initiatives, thus favoring the exchange and sharing of experiences on strategic health topics. It plays a key role in leveraging its ability to call on experts and high-level officials to collaboratively address these challenges. The WHO has been in charge of coordinating, organizing and keeping record of the group’s weekly meetings, as well as systematizing the advances and challenges presented in the different research sites.

#### Pan American health organization (PAHO)

PAHO/WHO in Brazil has had a central role in the technical conduct of the public health emergency by ZIKV, contributing to the MoH in the national response to this epidemic in Brazil and in the countries of the region, with the direct participation of its specialized teams in the field, mainly in the infection hotspots, supporting interventions in the areas of surveillance, epidemiological analysis and purchase of laboratory supplies. PAHO has facilitated the dialogue among several health managers in Latin America and contributed to the production of knowledge about ZIKV infection, both in Brazil and in the region. It has also played an important role in the management of funds earmarked for the development of strategic research activities [13].

#### Oswaldo Cruz Foundation (Fiocruz)

Fiocruz-RJ was responsible for coordinating the study and managing funds from international sources: Wellcome Trust, WHO and WRAIR. Additionally, it is in charge of carrying out laboratory tests together with FMT-HVD and Institute Aggeu Magalhães (CPqAM), in Fiocruz-PE, coordinating the laboratory management interface of the study with CGLAB/SVS. Fiocruz is a federal agency whose mission is to produce, disseminate and share knowledge and technologies aimed at contributing to the promotion of health and quality of life for the Brazilian population. Besides generating knowledge, Fiocruz is also responsible for the production of medicines, immunobiologicals and diagnostic tests [17], and works as a Collaborating Center for Global Health and South-South Cooperation, through its Center for International Relations in Health (CRIS Fiocruz) [18]. With the declaration of ZIKV as a Public Health Emergency of National Importance, Fiocruz prepared the “Fiocruz Plan” for coping with this public health emergency, presenting action strategies in several areas, including the national and international technical cooperation that supports the ZIKABRA study cooperation [17], a Zika social research network, among other initiatives.

#### Tropical Medicine Foundation Dr. Heitor Vieira Dourado (FMT-HVD)

FMT-HVD was the center responsible for the management of the national funding of the study. Patients from the city of Manaus, made up almost the entire sample of the study and the researchers were essential for the recruitment and follow-up of volunteer participants, laboratory analyses and transport of samples to the other sites in the national territory. As a national and world reference center for the treatment of tropical diseases, FMT-HVD plays an important role in clinical research and diagnosis and treatment of tropical diseases in the Amazon.

#### Wellcome Trust (WT)

The Wellcome Trust supports major projects in partnership with WHO and other institutions around the world to respond quickly to the global health threat caused by the ZIKV, including ZIKABRA. Its funding schemes offer grants across biomedical sciences, population health, medical innovation, humanities and social sciences, and public engagement [19].

#### The National Institutes of Health (NIH)

The NIH, more specifically the National Institute of Allergy and Infectious Diseases (NIAID), has partially funded the study through a grant, obtained via a bid notice. It promoted the research in areas such as the natural history of the disease, basic research on ZIKV, pathogenesis, rapid diagnostic tests, as well as treatments and vaccines [20].

#### Walter reed Army Institute of research (WRAIR)

In this cooperation, WRAIR added its experience by providing scientific resources as a center of excellence for immunology and the development of vaccines and medicines for diseases such as dengue, Zika, Ebola, coronaviruses, malaria, HIV/AIDS and others. WRAIR’s contributions to the ZIKABRA study included co-funding, protocol review, laboratory testing, technology transfer, laboratory capacity building and production and dissemination of results.

### Regulatory and governance context

The systematization and governance of the ZIKABRA Cooperation was based on the formalization of a memorandum of understanding (MoU) between the Ministry of Health, Fiocruz-RJ, WHO and WRAIR, which established the technical criteria for the cooperation, the allocation of responsibilities among the partners, the sharing of technical and scientific information and the commitment to national and international ethical and regulatory requirements [21]. In addition to ensuring sustainable training, the MoU was designed to create a local knowledge base and support future development of other programs with similar components [21]. It should be noted that the close collaboration among ZIKABRA scientists has led to the qualification of technicians at the post-doctoral, master’s and other levels. The MoU is complemented by a Term of Reference containing guidelines for disseminating the results of the study [21]. A Steering Committee followed up the products derived from the study and supported the dissemination of data relevant to public health.

Within the scope of the ZIKABRA Cooperation governance, a weekly dialogue routine was established since the study protocol development phase, as proposed by WHO, through the use of virtual platforms, in order to enable the participation of those involved, with transparency and integration of the study management as the ethos that characterizes its governance. In these meetings, events that occurred at each site, advances in recruitment, logistical problems faced, and the solutions found, doubts, decentralized monitoring of the protocol follow-up and other topics are reviewed. Each of these meetings is documented by WHO in minutes that record the topics covered, the decisions and courses of action and are circulated to those involved on a weekly basis. This routine helped everyone in the process of following up the work on different areas and sites.

In short, the programmatic instruments supporting the study are analyzed in this locus, namely: the MoU [21]; the research protocol; the Term of Reference for dissemination of the study scientific results; the mechanisms for planning and following up the study’s actions; the research sites life memory documentation; the technical reports of follow-up of the participating institutions; the compliance with the ethical aspects of research with human beings; the strategies for reaching consensus in the collective decisions of the research team and the set of participating actors; and the scientific production.

### Regulatory framework for ethics in research with human being

In Brazil, the National Commission for Research Ethics (CONEP) regulates research involving human beings and coordinates the institutions’ Research Ethics Committee (CEP) network, forming the CEP/CONEP System. The current regulatory framework is the CNS/MoH Resolution 466/2012 [22]. After the ZIKV emergency, the CNS Resolution No. 580 [23] was published, to allow research protocols, that are strategic for SUS (Brazil’s Unified Health System), to be processed as a matter of urgency, representing a considerable advancement for the agility of ethical evaluation in research, essential during health emergencies.

It is noteworthy that, in the ZIKV emergency context in Brazil, the CEP/CONEP System needed to adapt quickly to this reality [24] in order to appraise the large volume of research projects that have emerged, while attending to the relevant ethical and scientific foundations [22]. The ZIKABRA protocol was timely approved by CONEP and submitted for ethical evaluation at the local CEPs of the participating centers in order to comply with the double ethical evaluation required in the country. The process of ethical evaluation at the local and central level observed in the ZIKABRA’s case indicated the need for improvement in the CEP/CONEP System’s harmonization, considering that the CEPs of the participating institutions and services did not keep pace with CONEP to meet the requirements of the double ethical evaluation set out in the country.

The ZIKABRA study strictly followed the current regulations for the transportation, processing and use of human biological material, for the purpose of creating biorepositories and sending biological samples outside the country in accordance with national rules [25, 26]. For the internal management of the project, the MoU guarantees the sharing of human biological material stored in a biorepository and related information among partner institutions.

### ZIKABRA international scientific cooperation framework

**Fig. 1:**
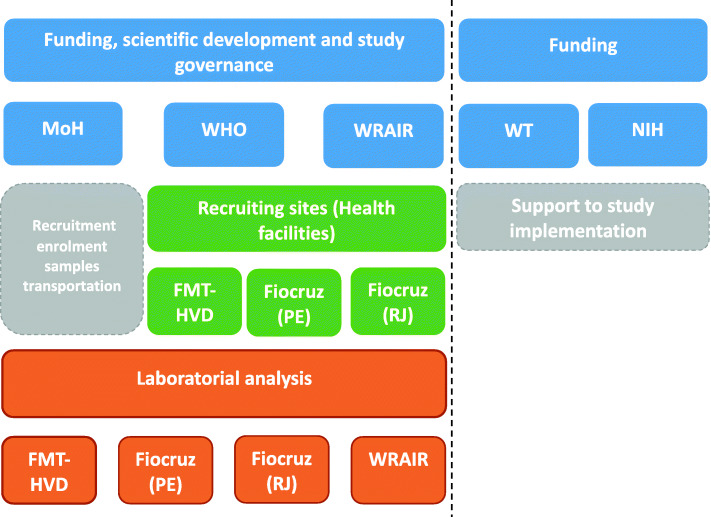
ZIKABRA International Scientific Cooperation Framework. *Source:* SPG

The ZIKABRA Cooperation (Fig. 1), is based on the partnership, support and promotion of public institutions of excellence, common funding for joint development and performance of their activities and future prospects for generating technological innovation such as the development of anti-ZIKV vaccines and specific diagnosis [21]. We consider that these features support a model of international scientific-technological cooperation [27].

This cooperation can be thought of as an example of global health governance, a process that makes up the type of collaboration between countries that work with common goals and interests. It can have a wide and flexible range of formal and informal joint activities that may include funding and coordination in health matters [28]. It can also operate at different levels of dialogue and foster the exchange of ideas on specific topics of expertise as one of the main challenges to be delivered.

The ZIKABRA Cooperation had a direct benefit for Brazil, but it also has potential applicability to other settings. The evidence generated by this cooperation could be used more broadly to address efforts regarding vulnerable and most at-risk population, such as those in countries with active transmission of the ZIKV or other emergent pathogens. It is, also, expected that the scientific information produced can support national and international guidelines for body fluid testing and contribute to the future health technology innovations [21].

However, we point out that one of the main barriers to researching in health emergency scenarios is the bureaucratic mechanisms and the slow chain procedures in conducting the rational use of financial resources in the implementation of the study. For instance, the delay in building the MoU, hiring qualified personnel, procurement and importing supplies, standardizing laboratory tests, transfer of funds to local sites, excessive delay in adjusting the work plan and to reach international quality standards.

It should be noted that the team of collaborators and supporters from the institutional partners played a leading role in the implementation of the ZIKABRA Study. The existence of this joint team of collaborators and supporters was key to overcome the barriers that came up during the throughout the execution of the study.

Working in cooperation in fact contributes to achieving more significant results than each group working in isolation. For this reason, careful structuring, relevant distribution of functions, well-coordinated collaboration and cooperation are pivotal. Cooperation also enables to provide consultation and coordination mechanisms so that this synergy takes place effectively [29].

### Lessons learned from the ZIKABRA international cooperation

Brazilian practices of international cooperation in health have shown fruitful results in tackling public health problems with the consolidation of research and development actions. Other studies have been produced showing the importance of international collaborations for responding to the ZIKV public health emergency, including innovation, joint publications, and research networks [30].

Another Zika research consortia were installed by the European Commission, for instance the Zika Preparedness Latin American Network (ZikaPLAN) [31] to address the research gaps in combating Zika and to establish a sustainable network with research capacity building in the Americas. This network involves the participation of 25 multinational and interdisciplinary institutional partners from Europe, Latin America, North America, Africa, and Asia. Created in October 2016, this consortium initiative includes 15 work packages in different areas: virology, diagnostics, entomology and vector control, modelling to clinical cohort studies in pregnant women and neonates, as well as studies on the neurological complications of Zika infections in adolescents and adults. Also, the ZikaPLAN diagnostics evaluation platform was rapidly used for evaluation of COVID 19 diagnostics [32].

It’s important to highlight the research protocols coordinated by WHO like the ZIKV Individual Participant Data (IPD) Consortium, that aims the development of systematic review to describe and gather data to IPD from longitudinal studies of pregnant women with ZIKV infection during pregnancy and fetal, infant or child outcomes, in the absence of a ZIKV vaccine or prophylactics [33, 34].

Another network initiative is supported by the European Commission (EC) Horizon 2020, was structured as a Consortium that comprises 53 partners organized in eight scientific and one management work packages (WPs), as well as three cross-cutting WPs, it’s the ZIKAlliance Consortium. This is a multicentre research that covers Latin America and the Caribbean region. Among the goals is to describe the dynamic course of the ZIKV epidemic in pregnant women (PW), children (CH) and natural history (NH) cohorts. This Consortium also collaborates with the EC H2020-funded consortia: ZikaPlan and ZikAction. It works as a spin-off for newly emerging infectious diseases, as now seen for COVID-19 [35].

It is relevant to highlight that for all rare disease outcomes in ZIKV infection, networking has been a concrete response to share action regarding side events such as Guillain Barre Syndrome and other neurological complications, early postnatal symptomatic ZIKV infections and other rare events such as thrombocytopenia. Even more frequent events such as attack rates and sexual transmission [7] can be best documented in meta-analyses of large cohorts. For instance, the International Guillain-Barré syndrome Outcome Study (IGOS) network or GeoSentinel, a network of travel medicine providers that investigates returning travelers [36–39].

We understand that organizational (regarding global public health emergency response), administrative and regulatory processes must be sufficiently agile to meet the demands of scientific research in favor of the benefits that science can bring to public health [40], without neglecting the good research practices.

In ZIKABRA study, groundwork was done in the health units of the selected sites, which involved the participation of health service coordinators, doctors, nurses and social workers, through prospecting visits and meetings with the presence of the researchers in charge and members of WHO, PAHO, WRAIR and MoH. During these visits, technical training on the protocol, good research practices, ethical respect for volunteer participants, structuring of services to avoid interruption in the routine care to regular patients, professionalization of non-academic professionals, among other activities, were promoted. The results were particularly enriching due to the experience shared between the site health teams and the ZIKABRA team, favoring the engagement of all actors involved for the good execution of the study.

One of the main bottlenecks in the operationalization of the ZIKABRA study was the change in ZIKV epidemiology, making it impossible to recruit participants in Rio de Janeiro, and very few participants in Recife. Additionally, there was a mismatch between the finalization of the ZIKABRA protocol, including all its regulatory and financial aspects completed in January 2017, and the end of the Rapid Action Strategy by SVS/MoH, on October 31, 2016, as well as the PHEIC closure by WHO, on November 18, 2016 [9]. This demanded an operational logistics rearrangement in the laboratories of the CPqAM - Fiocruz-PE, and Fiocruz-RJ, and in the support of the laboratory of the FMT-HVD.

On the other hand, the ZIKABRA Cooperation structure, with clearly defined roles in the operational instruments, has overcome the difficulties encountered, reaching the proposed goals, greater efficiency in fund management and strengthening research autonomy. The ZIKABRA study leaves as a legacy the interaction among the actors experienced in an arena of weekly discussions where decisions were made in full respect for the cooperative ethos among peers, the intertwining of institutional visions, in order to support the team of collaborators, aiming at the common interest and achieving excellence in results.

Based on this cooperation, it is suggested that there is greater flexibility and autonomy in the mechanisms of disbursement of funds and execution of the work plan, ease in hiring qualified human resources and in the purchase and import of laboratory supplies, standardization of diagnostic tests, among others. Table 1 summarizes the lessons learned from the ZIKABRA cooperation.

**Table 1:** Lessons learned

Topic	Challenges	Proposed solutions
Ethical evaluation in local and international partner institutions	Different times for ethical evaluation of the study in the different national and international ethical instances	Harmonize the CEP/CONEP System for urgent ethical evaluation in the different instances, respecting the Good Research Practices, in the national context.
Multi-institutional funding	Raising and making public funds available quickly by partner institutions	Fine-tune regulatory and decision-making processes at partner institutions and make funds available on an urgent basis.
Technology availability	Standardization of diagnostic tests throughout the study execution period	Promote the sustainability and continuity of technological innovation development activities, training of high-level human resources.
Formalization of operational instruments	Sharing of institutional and individual responsibilities among the different actors	Expedite the drafting and harmonization of the basic text of the agreement among the parties.

## Conclusions

The legacy of the ZIKABRA Cooperation includes the construction of installed research capacity in the site, with improvement of laboratory equipment, laboratory and clinical data management platforms, training of academic and non-academic human resources, dissemination of generated knowledge, advancement of knowledge about the studied theme, knowledge transfer, availability of the ZIKABRA study protocol for development of similar studies, thus favoring the collective construction of knowledge to provide public health emergency responses. Additionally, the benefits arising from the study contribute to the strengthening of new partnerships with national and international researchers of recognized excellence in sciences, supported by public and government institutions in political, economic and technical terms [24].

We highlight some steps towards the potential continuity of the ZIKABRA Cooperation initiative, such as the idea of WHO as a global health hub mobilizing experts and putting its tradition, prestige and knowledge to the services of technical responses achieving health goals and sharing understanding and capabilities about the actions, norms and procedures adopted by the set of participating institutions.

Another relevant lesson learned is the horizontal cooperation among Brazilian researchers and institutions all over the country. The ZIKABRA project provided [41] technical responses to clinical and laboratory questions of the research and also generated indirect benefits in the arena of policy studies and health cooperation frameworks.

It would also be important to bear in mind the role of interaction among government bodies of the participating institutions that takes place in the joint follow-up of the sites and project performance. Field supervision of all stakeholders during the implementation of the project occurred periodically. This helped to share the level of knowledge of the site settings by all participants and to avoid institutional constraints.

## Data Availability

Data underlying the study cannot be made publicly available due to ethical concerns, as data contain several personally identifiable information. Data are available from Oswaldo Cruz Foundation for researchers who meet the criteria for access to confidential data. Contact information: Institutional Ethics and Research Committee of the Evandro Chagas National Institute of Infectious Diseases, email: cep@ini.fiocruz.br or Guilherme Amaral Calvet; email: guilherme.calvet@ini.fiocruz.br.

## Abbreviations

ZIKV: Zika virus
WHO: World Health Organization
DCCI: Department of Chronic Condition Diseases and Sexually Transmitted Infections
SVS: Health Surveillance Secretariat
MoH: Ministry of Health
ZIKABRA Study: Study on the persistence of Zika virus (ZIKV) in body fluids of patients with ZIKV infection in Brazil
PHEIC: Public Health Emergency of International Concern
FMT-HVD: Tropical Medicine Foundation Dr. Heitor Vieira Dourado
Fiocruz: Oswaldo Cruz Foundation, RJ
CPqAM – Fiocruz – PE: Institute Aggeu Magalhães/Oswaldo Cruz Foundation
WRAIR: United States Department of Defense laboratory, and Walter Reed Army Institute of Research
WT: Wellcome Trust
NIH: National Institutes of Health
PAHO: Pan American Health Organization
CGDEP/SVS: General Coordination for the Development of Epidemiology in Services/ Health Surveillance Secretariat
CGLAB/SVS: General Coordination of Public Health Laboratories/ Health Surveillance Secretariat
CRIS/Fiocruz: Cooperation, through its Center for International Relations in Health/ Oswaldo Cruz Foundation
CONEP: National Commission for Research Ethics
CEP: Research Ethics Committee
CNS/MoH: National Health Council/ Ministry of Health
SUS: Unified Health System
